# Big Role of Small RNAs in Female Gametophyte Development

**DOI:** 10.3390/ijms23041979

**Published:** 2022-02-10

**Authors:** Mohammad Aslam, Beenish Fakher, Yuan Qin

**Affiliations:** 1 Guangxi Key Lab of Sugarcane Biology, State Key Laboratory for Conservation and Utilization of Subtropical Agro-Bioresources, College of Agriculture, Guangxi University, Nanning 530004, China; aslampmb1@gmail.com; 2 Center for Genomics and Biotechnology, College of Agriculture, Fujian Agriculture and Forestry University, Fuzhou 350002, China; beenishfakher@icloud.com

**Keywords:** gametophyte, microRNA, short interfering RNAs, meiosis

## Abstract

In living organisms, sexual reproduction relies on the successful development of the gametes. Flowering plants produce gametes in the specialized organs of the flower, the gametophytes. The female gametophyte (FG), a multicellular structure containing female gametes (egg cell and central cell), is often referred to as an embryo sac. Intriguingly, several protein complexes, molecular and genetic mechanisms participate and tightly regulate the female gametophyte development. Recent evidence indicates that small RNA (sRNA) mediated pathways play vital roles in female gametophyte development and specification. Here, we present an insight into our understanding and the recent updates on the molecular mechanism of different players of small RNA-directed regulatory pathways during ovule formation and growth.

## 1. Introduction

**Figure 1:**
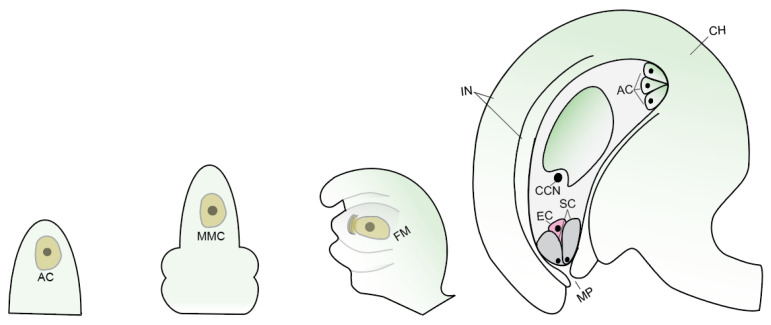
Female gametophyte development in *Arabidopsis*. AC—archesporial cell; MMC—megaspore mother cell; FM—functional megaspore; MP–micropyle; EC—egg cell; SC—synergid cells; CCN—central cell nucleus (2n); AC—antipodal cells; IN—integuments; CH—chalaza.

Flowering plants follow a life cycle alternating between the diploid (sporophytic) and haploid (gametophytic) generations (or phases) [1]. Generally, the diploid sporophytic tissue makes up the main body part, whereas the haploid gametophytes reside in specialized floral organs [2]. The mature male gametophyte (pollen) contains the male gamete in the form of two sperm cells, and the female gametophyte contains the female gamete. In most plants, the female germline starts with a somatic hypodermal cell that differentiates and forms a megaspore mother cell (MMC), the first committed cell of the female germline lineage. After meiosis, MMC advances into a haploid female gametophyte, which develops into a mature ovule after cell differentiation and proliferation. The mature female gametophyte contains seven specialized cell types: one egg cell, one central cell, two synergid cells, and three antipodal cells (Figure 1) [2,3]. Fertilization, which takes place in the female gametophyte of the flower, is a crucial turning point in the alternation of generations of angiosperms. A fusion of male and female gametes occurs during fertilization once the sperm cells are delivered to the embryo sac by the pollen tube. The egg and central cell separately fuse with sperm cells and develop into embryo and endosperm, respectively, and eventually, the mature seeds are formed [1].

During its lifespan, the plant genome produces numerous regulatory sRNAs, typically 21–24 nucleotide (nt) in size, which mainly regulates gene expression [4]. They function in many biological processes, including development, reproduction, stress response, silencing repetitive elements, and against viral infections [5,6]. Generally, sRNAs are derived from the processing of double-stranded RNA (dsRNA) intermediates (hairpin precursors) or via the synthesis of dsRNA from the single-stranded RNA (ssRNA) template by the activity of RNA-DEPENDENT RNA POLYMERASES (RDRs) [7]. These longer precursors of double-stranded duplex RNAs are further processed by endonuclease DICER-LIKE (DCL) proteins [8]. The DCL activity on the dsRNA duplex results in the generation of small RNA duplexes, one strand of which associates with Argonaute (AGO) proteins. Subsequently, the sRNA-loaded AGO hybridizes with target RNAs resulting in the cleavage, degradation, or translational repression of the target depending on the sequence complementarity and partner AGO protein [4,7].

Recent evidence suggests that sRNAs play critical roles in gametophyte development [9,10]. Here we review the functions of different sRNA components and recent progress in understanding their role in female gametophyte development, focusing on *Arabidopsis*.

## 2. Small RNA Processing and Biogenesis

### 2.1. MicroRNAs

**Figure 2:**
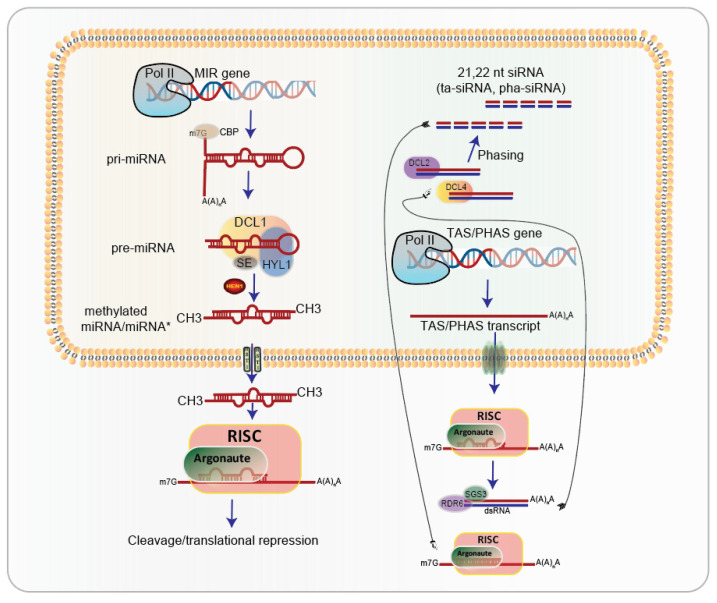
The biogenesis of small RNA in plants. The microRNA (miR) genes (left panel) are transcribed into primary microRNAs (pri-miRNAs) by RNA Polymerase II. The hairpin-like secondary structure is further processed into mature miRNAs. Processing or pri-miRNA requires two sequential cleavages by DICER-LIKE 1 (DCL1) and is assisted by RNA-binding proteins, which recognize different parts of pri-miRNAs and /or associate with key components of the processing complex. After their production, miRNA/miRNA* duplex is methylated by methyltransferase HEN1 and then transported out of the nucleus by HST1. In the cytoplasm, miRNA strand is loaded into ARGONAUTE 1 (AGO1) containing RNA-induced silencing complex (RISC), which is then guided to target mRNA. The ta-siRNA/pha-siRNA biogenesis (right) involves the targeting of a Pol II generated long non-coding TAS/PHAS transcript by miRNA-guided AGO cleavage, followed by the respective synthesis (by RDR6) and stabilization (by SUPPRESSOR OF GENE SILENCING 3; SGS3) of dsRNAs. These dsRNAs are processed by DCL4/DCL2 and loaded into AGO1 proteins for PTGS as detailed for miRNA.

Based on the differences in their biogenesis and targeting mechanisms, the plant sRNA population has been broadly categorized into two major groups: microRNAs (miRNAs) and short interfering RNAs (siRNAs) (Figure 2) [11]. Plant miRNAs (generally 21 nucleotides (nt) in length) are transcribed by RNA Polymerase II (Pol II) from endogenous genes with large primary microRNAs (pri-miRNAs) transcripts. The single-stranded pri-miRNA molecule folds back and forms an imperfect hairpin structure which is recognized by endonuclease DCL1. The sequential processing of pri-miRNA by DCL1 and other accessory proteins generates the mature miRNA duplex, known as miRNA/miRNA* [12]. One of the strands of the duplex is then loaded to the AGO protein forming the RNA induced silencing complex (RISC); the other strand of the duplex is generally degraded (for details, see [7]). The RISC then directs the post-transcriptional regulation of mRNA or other non-coding RNAs depending on the base pair complementarity among the target RNA and the miRNA [12,13]. In some cases, the complex triggers the generation of siRNAs (Figure 2).

### 2.2. Short Interfering RNAs

In contrast to miRNAs, the precursors of plant siRNAs are long dsRNAs produced in various ways, such as the hybridization of sense and antisense transcripts, fold back of inverted repeat sequences, sequence complementarity between unrelated RNA molecules and RDRs activity [4]. The siRNAs are also generated by the DCL/AGO system; however, unlike miRNAs, they do not possess the precisely processed stem-loop precursor. Additionally, siRNAs target transcripts that are generally the loci of their origin, whereas plant miRNAs target different RNAs rather than their precursor transcripts. Typically, DCL2, DCL3, and DCL4 process the endogenous plant siRNAs. Plant siRNAs are categorized into two significant classes (1) secondary siRNAs and (2) heterochromatic-siRNA (het-siRNAs). Secondary siRNAs are further divided into different sub-classes, such as trans-acting small interfering RNAs (ta-siRNAs), phased small interfering RNAs (pha-siRNAs), epigenetically-activated small interfering RNAs (ea-siRNAs), and natural antisense (nat-siRNAs).

The biogenesis of secondary siRNA, such as pha- and ta-siRNA requires a primary interaction of AGO-miRNA or AGO-siRNA with target RNA. The sliced target product of this interaction is then converted into dsRNA by the activity of RDR6. The resulting intermediate dsRNA is further processed into siRNA duplexes either by DCL4 (to generate 21 nt long duplexes) or DCL2 (to generate 22 nt long duplexes). Often, the first nucleotide of secondary siRNA begins at every 21 or 22 nucleotides from the cleavage site, a phasing process, and the subsequent siRNAs are known as pha-siRNAs [14,15]. The newly created secondary siRNAs then bind to AGO proteins and target the transcripts, including the original transcript depending on sequence complementarity. This feedback loop results in a reliable gene silencing mechanism [5]. The secondary siRNAs that target the mRNAs distinct from their precursor RNA are referred to as trans-acting siRNAs (ta-siRNAs) [16].

Interestingly, ta-siRNAs biogenesis is an example of crosstalk between different sRNA pathways. In *Arabidopsis*, ta-siRNAs biogenesis begins with transcription of one of the four TAS loci into long non-coding RNA transcripts by RNA Pol II [16,17]. The TAS transcript further gets targeted and cleaved by AGO-miRNA complex (miR173-AGO1 for TAS1 and TAS2, miR390-AGO7 for TAS3 and miR828-AGO1 for TAS4), reviewed by [17]. The SUPPRESSOR OF GENE SILENCING 3 (SGS3) binds and stabilizes the cleaved TAS transcript fragments [16,18,19]. The RNA fragments serve as a template for dsRNA synthesis by RDR6 and subsequent siRNA production by DCLs processing (Figure 2).

**Figure 3:**
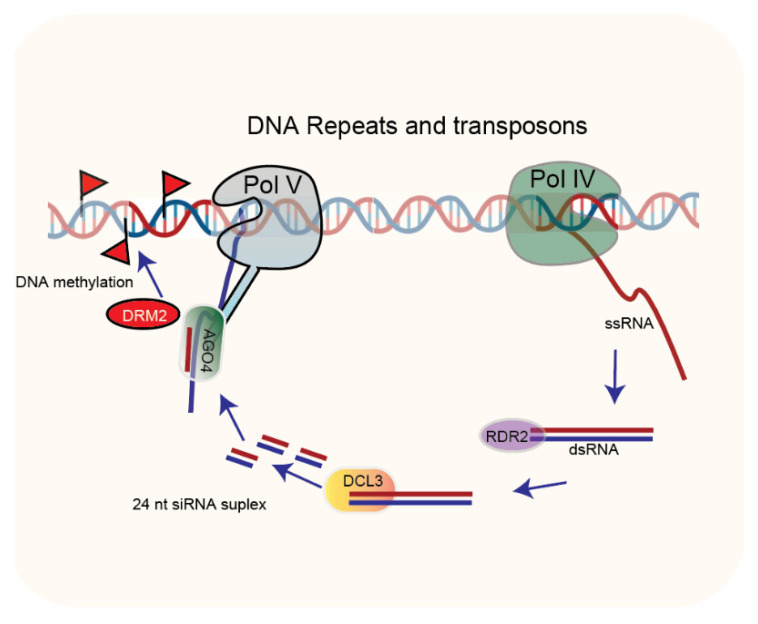
Pol IV initially transcribes DNA repeats and transposons. RNA-dependent RNA polymerase 2 (RDR2) then produces dsRNAs, DCL3 to process dsRNAs to 24-nucleotide siRNAs, which are stabilized by methylation on their 3′-terminal 2′-O-methyl group that is introduced by HEN1 and then loaded onto AGO. The AGO4 recruits DOMAINS REARRANGED METHYLTRANSFERASE 2 (DRM2) during Pol V mediated transcription, responsible for de novo methylation at the homologous genomic sites, resulting in transcriptional gene silencing (TGS).

Plant het-siRNAs are the most abundant small RNAs that differ in biogenesis and function from miRNAs and secondary siRNAs. During het-siRNAs biogenesis, the Pol IV transcribed RNA serves as a template of RDR2 for dsRNA synthesis. The DCL3 then processes the dsRNA into a 24-nucleotide-long siRNA duplex (Figure 3). The het-siRNAs have not been reported for the targeting of mRNA; instead, they mediate transcriptional silencing through RNA-directed DNA methylation (RdDM) of transposons and pericentromeric repeats [7,20,21]. Another class of endogenous sRNAs, PIWI-interacting RNAs (piRNAs, 25–29 nt length), are produced in animals that arise from clusters of repetitive DNA in a Dicer independent mechanism. piRNAs are not described in plants.

## 3. Argonautes (AGOs)

Argonaute proteins play a central role in the small RNA-mediated regulatory pathways by interacting with a variety of sRNAs, including miRNAs and siRNAs, which are essential for eukaryotic developmental pathways [22]. The AGO family in plants consists of multiple diverse members; for example, the *Arabidopsis* genome encodes ten AGO proteins, all of which contain PAZ, MID (middle), and P-ELEMENT-INDUCED WIMPY TESTIS (PIWI) domains [22,23]. Each of the three domains of the AGO protein has a specific function in the small RNA pathway. The PAZ domain recognizes the 3′ end of small RNAs and MID domain binds to the phosphate at 5′ end of small RNA. At the same time, the PIWI domain possesses endonuclease activity [24]. *Arabidopsis* AGO proteins have been divided into three groups based on their phylogenetic relationship and small RNA binding capacity. The first clade contains AtAGO1, AtAGO5, and AtAGO10; the second clade members include AtAGO2, AtAGO3, and AtAGO7 and the remaining AtAGO4, AtAGO6, AtAGO8, and AtAGO9 are within the third clade [22]. However, some reports have classified AGOs in four clades separating AGO5 from clade 1 [25]. The existence of multiple members in a clade suggests possible functional redundancy among the same clade members [13]. For example, the third clade members (AGO 4/6/8/9) are referred to as RNA-directed-DNA-methylation (RdDM) AGOs because of their involvement in RdDM [26]. AGO4, the most studied member of this clade, is responsible for accumulating a subset of secondary heterochromatic siRNA (Figure 3) [27]. AGO4 also controls histone methylation by recruiting DNA methyltransferase DRM2 at specific loci (Figure 3) [27,28]. Additionally, AGO4 participates in the plant immunity response against bacteria, viruses, and fungi [29,30,31]. AGO6 is believed to possess partial redundant function with AGO4 as it also plays critical roles in siRNAs accumulation, DNA methylation, and transcriptional gene silencing (TGS) at specific loci [32].

## 4. Small RNA Machinery and Female Gametophyte Development

MicroRNAs (miRNAs), such as miR167, regulate ovule morphogenesis and fertility by limiting the expression levels of their target genes AUXIN RESPONSE FACTORS (*ARFs*) [33,34]. By inhibiting the expression of ARF6 and ARF8, miRNA167 acts as a positive regulator for ovule development. Besides, the defects of ovule development in the *mir167* mutant can be rescued by introducing either *arf6* or *arf8* [35]. Furthermore, a significant reduction in the accumulation of miR167 is observed in *hen1* mutants resulting in ectopic expression of *ARF6* and *ARF8* and introduction of *arf6; arf8* failed to rescue the ovule defects in *hen1* mutant [36,37,38]. Consistently, miR165/6 targets *Class III HOMEODOMAIN-LEUCINE ZIPPER* (*HD-Zip III*) gene *PHABULOSA* (*PHB*) [39]. Similar to its mode of action in embryos, root, and leaf primordia, miR165/6 acts non-cell-autonomously to spatially regulate the *PHB* expression pattern in developing ovules [39,40]. PHB transcripts localize to the distal chalazal region in the early ovule primordium. During the integument initiation, miR165/6 restricts *PHB* transcripts to the inner layer of the inner integument [39,41]. In contrast, miR165/6-resistant gain-of-function *phb-1d* transcripts accumulate *PHB* in both the inner and outer layers of the inner integument. While in heterozygous PHB/*phb*-*1d* plants, ovule development is arrested due to defective outer integument development [41,42]. These observations suggest that precise control of the PHB expression by miR165/6 is essential for ovule morphogenesis. Besides, mutants of microRNA biogenesis machinery such as HEN1 and HYL1 show defective ovule development. The ovules in these mutants have abnormal embryo sacs due to defects in asymmetric integument growth and compromised pollen tube guidance resulting in reduced female fertility [38]. These findings clearly indicate the participation of miRNA machinery in ovule development.

Previous studies have indicated that AGO proteins play crucial roles in gametophyte development. Consistently, some AGO proteins express preferentially in reproductive organs and get enriched in germline cells where they are believed to function in the meiotic processes and specification of the cell fate [43,44,45,46]. For example, Olmedo-Monfil et al. (2010) noticed several transcriptomic data showed high expression of *AGO9* in ovules and anthers. They also found the expression of *AGO9* mRNA throughout ovule development [46]. Using several biochemical and genetic approaches, the authors demonstrated that the mutations in *AGO9* led to the differentiation of multiple gametic cells that can initiate gametogenesis. AGO9 protein gets localized in the cytoplasmic foci of somatic companion cells instead of the gamete lineage, where it preferentially interacts with transposable elements (TEs) derived 24 nt sRNAs, an obligatory step to silence TEs. In an extended study, the authors further showed that these 24 nt sRNAs mainly correspond to retrotransposons (Athila, Gypsy CACTA, and less frequently LINE or mutator) and are preferentially expressed in the ovule before pollination. Besides, in the ovule, AGO9 is required for the inactivation of a large number of long terminal repeat retrotransposons (LTRs), and its primary TE targets are found in the pericentromeric regions of all five chromosomes, implying a link between the AGO9-dependent sRNA pathway and heterochromatin formation [46,47]. Collectively this indicates that AGO9 regulates female gamete formation by limiting gametophyte precursor cell specification in a dosage-dependent and non-cell-autonomous way. Moreover, in maize, a mutation in AGO104, a homolog of *A. thaliana* AGO9 explicitly expressed in somatic cells surrounding the female meiocyte, results in the failure of MMC meiosis but not multiple MMC-like cells [44].

The clade one members, AGO1, AGO5 and AGO10, are generally involved in microRNAs related to PTGS and have been implicated in shoot meristem maintenance [12,48]. AGO1 is extensively studied among this clade, and mutants of *ago1* display pleiotropic phenotypes due to its involvement in miRNA and tasi-RNA pathways [22,49]. The other member of this clade, AGO5, binds 21 nt, 22 nt, and 24 nt siRNAs with a 5′C bias and interacts with *miR156* to modulate the expression of *SPL* transcription factors [50,51] resulting in an early flowering phenotype [50]. The semi-dominant mutant of *ago5* causes defects in the initiation of mega-gametogenesis [52]. *AGO5* expresses in the megaspore cells and the somatic cells neighbouring megaspore mother cells [52,53]. Moreover, rice *AGO5* expresses during all the stages of flower and seed development [43], and a mutation in AGO5 clade member MEIOSIS ARRESTED AT LEPTOTENE 1 (MEL1) frequently shows abnormal tapetum and dysfunctional pollen mother cells (PMCs) in anthers [54,55]. MEL1 mutation results in early meiotic arrest resulting in male sterility; however, the null mutant of *Arabidopsis ago5* does not show a sterile phenotype suggesting a possible convergent evolution pathway in monocots. Although MEL1 participates in anther development, its expression in ovules specifies the unidentified role of MEL1 during gametophyte development. AGO10 attenuates miR165/6 activity by sequestering the miR165/6 to promote its degradation by SDN1 and SDN2 [56,57]. In AGO10 immunoprecipitation, miR398 was the second most abundant miRNA species implicating that AGO10 could also sequester miR398. Following this hypothesis, Cai et al. uncovered the critical roles of AGO10 and miR398 in female gametophyte development [9,57]. The authors showed that *AGO10* expresses in the chalaza of ovules from stage 2-V to stage 3-VI, where AGO10 sequesters miR398 to maintain its target (AGL genes) in the female gametophyte during ovule development. Thus, acting as a gatekeeper, AGO10 controls miRNA movement between the female gametophyte and sporophytic tissues [9]. The other clade, AGO2, AGO3, and AGO7, seemed to have more specialized functions. AGO7 was implicated in accelerating vegetative phase change and producing slightly abnormal flowers with no other apparent effects on shoot morphology [58].

**Figure 4:**
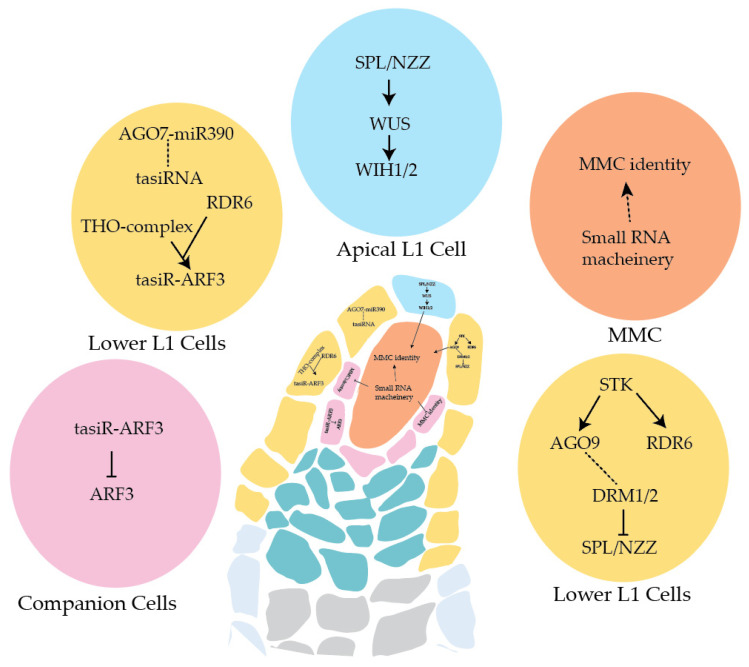
Summary of key pathways involved in female germline differentiation. The distinct processes in the megaspore mother cell (MMC, orange), the companion cells (pink), the apical L1 cell (blue) and the lower L1 cells (yellow) are represented in the *Arabidopsis* ovule. The cell-specific small RNA-dependent pathways are required to develop MMC fate and ovule development.

Throughout plant growth and development, ta-siRNAs play indispensable roles [15,59,60]. Mutants of ta-siRNA pathway proteins such as *sgs3* and *rdr6* exhibit multi numerary MMC phenotypes [46,61]. An EMS-based genetic screen on *rdr6* mutant by Su et al. (2017) identified that *j66* mutant enhanced *rdr6* phenotype and showed multiple MMC-like cells in pre-meiotic ovules [62]. Further characterization of the *j66* mutant revealed the mutation located in the TEX1 gene, encoding for one of the components of the THO/TREX complex [62]. The THO complex is implicated in the biogenesis of the mRNA and endogenous/exogenous siRNA production [63,64,65,66]. THO complex also functions in the repression of the MMC fate of the surrounding tissues [62]. The double mutant *rdr6 tex1* displays an increased number of MMC-like cells compared to *tex1* or *rdr6* alone, implying that the THO complex and the RdDM pathway work together to suppress MMC fate. The AGO7-miR390 complex functions in tasiR-ARF production by targeting long non-coding RNAs generated from the TAS3 loci [67,68]. These tasiR-ARFs are so named because they target several ARFs, and one of the ARF, ARF3, is crucial for ovules integument development [69]. In both *tex1* and *rdr6* mutants, the expression of tasiR-ARF was reduced, and the mutants with reduced tasiR-ARF levels such as *rdr6*, *tex1*, and *tas3* exhibit a multiple MMC-like cell phenotype. Moreover, the double mutant of *arf3tex1* is rescued to wild-type levels, demonstrating that the control of MMC differentiation by the THO complex is regulated by the repression of ARF3 via tasiR-ARF [62]. Consistently, the ectopic expression of *ARF3* leads to the multiple-MMC formation suggesting the involvement of AGO7-miR390 mediated regulation of *ARF3* during early ovule development [62]. This hypothesis was recently verified, and it was shown that the mutants of AGO7, *zip-2* and *ago7-9,* show approximately 22 to 25 of multiple MMC-like cells in ovule primordia, validating that indeed the AGO7 mediated spatiotemporal regulation of *ARF3* is essential for ovule development (Figure 4) [10].

Moreover, mutants of the RNA-dependent DNA methylation (RdDM) pathway have been linked to restricting female germline to a single nucellus cell (Table 1). Multiple MMC-like cells are observed in plants lacking the classical RdDM components such as DCL3, Pol IV/Pol V and RDR2 [46]. Consistently, (siRNA)-dependent RdDM pathway proteins DOMAINS REARRANGED METHYLASES (DRM1 and DRM2) double mutant, *drm1drm2,* exhibit a similar phenotype to that of *ago9-2* and *rdr6-11* lines and show the formation of multiple MMC-like cells confirming the role of methylation in ovule development [61]. Notably, *rdr6*, the non-canonical RdDM RDR, also possesses the extra numeracy MMC-like cells in the nucellus [46,61]. In *Arabidopsis*, the MADS-box transcription factors SEEDSTICK (STK) and SHATTERPROOF (SHP1 and SHP2) redundantly control ovule identity [70,71,72]. STK expresses in the sporophytic cells of an ovule such as integuments, chalaza and nucellus and plays essential functions in ovule and seed development [61,73]. Surprisingly, in the ovules of *stk* mutant, the expression of *SPOROCYTELESS/NOZZLE* *(SPL/NZZ)* is found upregulated, whereas a reduction in the expression of *RDR6* and *AGO9* was observed [61]. It is found that STK directly binds the regulatory region of *AGO9* and *RDR6* to promote their expression solely in the lower cells of the L1 layer. Following ovule initiation, SPL/NZZ plays indispensable roles in MMC differentiation as exhibited by the *spl/nzz* mutant in which approximately 99% of ovules do not form MMC [74,75]. The RdDM pathway is essential to limit *SPL/NZZ* expression in the nucellus and confine it to the L1 cells at the top of the nucellus [61]. Consequently, the above findings establish that the mutations in the RdDM pathway genes, including *AGO9,* result in an ectopic expression of *SPL/NZZ*, which most likely accounts for the formation of the multi numerary MMC cells.

Furthermore, WUSCHEL (WUS), the homeodomain transcription factor, acts downstream *SPL/NZZ* and plays an essential role in assuring the MMC differentiation. As a result, *wus* mutants show multiple defects, including the lack of MMC-containing reproductive structures. It is now established that WUS regulates the expression of *WINDHOSE 1* (*WIH1*) and *WIH2*, and the absence of these two redundantly acting genes leads to loss of the MMC [76]. The expression of both the *SPL/NZZ* and *WUS* prior to germline initiation suggests that both are crucial for preparing the cell for germline formation [77].

## 5. Concluding Remarks

Since discovering the roles of eukaryotic sRNAs in RNA interference, different types of endogenous sRNAs, including miRNAs and siRNAs, have been characterized. After associating with Argonaute proteins, these sRNAs regulate nearly every major biological activity. Consistently, various sRNA pathways have been implicated in regulating gametophyte development. Besides, several members of the sRNA pathways are involved in female gametophyte development (Table 1). This specificity may have been elevated due to the complexity in sRNA populations, which further validates the functional diversity among different sRNAs. MMC number is restricted to one in ovules, and multiple MMC phenotype is often observed in sRNA mutants. Genes such as *RDR6* and *AGO9* inhibit somatic cells from attaining germline identity. Further studies may help find the new sRNA pathway genes that may confer MMC identity. The advancement in microscopy, CRISPR, single-cell transcriptomics and availability of cell-specific markers may facilitate the identification of undefined genes in the future.

## Figures and Tables

**Table 1:** List of sRNA pathway components involved in *Arabidopsis* female germline differentiation and development.

Name	Type	Target	Location in Ovule	Ref.
miR167	MicroRNA	*ARF6, ARF8*	Companion cells	[35,36,37,38]
miR165/6	MicroRNA	*PHABULOSA*	Integuments	[39,40]
miR390	MicroRNA	TAS3 loci	Lower L1	[62]
miR398	MicroRNA	*AGL* genes	Ovules at stage 3-VI	[9]
HEN1	Methyltransferase	miRNA/siRNA	Integuments	[38]
taSiR-ARF3	siRNA	*ARF3*	Lower L1, Companion cells	[62]
SGS3	Unknow	TAS transcript fragments	Lower L1	[46]
TEX-1	Component of THO/TREX	endogenous/exogenous siRNA production	Lower L1	[62]
DRM1/2	Methylase	DNA methylation of TEs	Lower L1 Cells	[61]
RDR6	RNA Polymerase	RNA Fragments	L1 layer	[46,61]
AGO10	Argonaute	miR398	Chalaza	[9]
AGO9	Argonaute	24 nucleotide (nt) sRNAs	L1 layer	[46]
AGO7	Argonaute	miR390	Distal Nucellus	[62]
AGO4	Argonaute	DRM2	L1 layer	[27,28]
STK mediated RdDM	RdDM	*SPL/NZZ*	Apical L1 layer	[61]
STK	MADS-box TF	*AGO9* and *RDR6*	Lower L1 layer	[61]
